# A Novel Human Respiration Pattern Recognition Using Signals of Ultra-Wideband Radar Sensor

**DOI:** 10.3390/s19153340

**Published:** 2019-07-30

**Authors:** Seong-Hoon Kim, Zong Woo Geem, Gi-Tae Han

**Affiliations:** 1 Department of Computer Engineering, Gachon University, Seongnam 13120, Korea; 2 Department of Energy IT, Gachon University, Seongnam 13120, Korea

**Keywords:** CNN, ultra-wideband radar, respiration pattern, pattern recognition, sleep quality

## Abstract

Recently, various studies have been conducted on the quality of sleep in medical and health care fields. Sleep analysis in these areas is typically performed through polysomnography. However, since polysomnography involves attaching sensor devices to the body, accurate sleep measurements may be difficult due to the inconvenience and sensitivity of physical contact. In recent years, research has been focused on using sensors such as Ultra-wideband Radar, which can acquire bio-signals even in a non-contact environment, to solve these problems. In this paper, we have acquired respiratory signal data using Ultra-wideband Radar and proposed 1D CNN (1-Dimension Convolutional Neural Network) model that can classify and recognize five respiration patterns (Eupnea, Bradypnea, Tachypnea, Apnea, and Motion) from the signal data. Also, in the proposed model, we find the optimum parameter range through the recognition rate experiment on the combination of parameters (layer depth, size of kernel, and number of kernels). The average recognition rate of five breathing patterns experimented by applying the proposed method was 93.9%, which is about 3%~13% higher than that of conventional methods (LDA, SVM, and MLP).

## 1. Introduction

There are a variety of existing methods for human sleep testing as a means of diagnosing diseases and improving sleep quality. Sleep analysis methods that are mainly used for disease diagnosis are performed through polysomnography. Recently, in the field of health care, sleep-breath analysis has been studied to improve sleep quality.

Polysomnography measures several items through sensors and devices. The basic items are sleep phase, arousal, respiratory flow, respiratory ability, and blood oxygen saturation concentration [1,2]. The devices with sensor used to measure these items are either directly attached to the body or measured through the oral and nasal cavities. Therefore, performance of polysomnography is possible only at facilities equipped with such devices. As a result, the environment in which the user normally sleeps is changed, so that it may be difficult to accurately retrieve data due to a shallow sleep or a bad sleep. To minimize these problems, portable sleep inspection devices (PSG Type II and III) are being used, but they can also affect sleep because they attach multiple sensors (nasal pressure transducer, chest belt, etc.) to the body [3,4,5]. Therefore, when collecting breathing-related information, non-contact sensors can be used to reduce sleep disturbance factors rather than conventional methods.

In the current healthcare field, research is underway to analyze the quality of sleeping with the detection of sleep apnea or snoring using various sensors [6,7,8,9]. However, the healthcare sector analyzes the sleep using less information than polysomnography, so the information provided to users is limited. Therefore, various data acquisition methods are required for accurate sleep quality analysis. In recent years, devices for measuring data with a non-contact type sensor have emerged for the user’s convenience rather than contact type sensors.

The UWB (Ultra-WideBand) sensor, which has been used as a method for wireless communication in the past, has recently been recognized as a non-contact sensor and implemented as a radar system, which is used in healthcare research as well as obstacle detection [10,11,12,13,14]. Especially, it is known that UWB Radar can detect respiratory and pulse signals due to its signal characteristics. UWB radar has been used to detect apnea and respiratory rate [15,16,17,18,19]. However, in order to be eventually used for polysomnography or healthcare, it is necessary to recognize different breathing patterns as well as to measure the number of breaths per minute or detect the apnea.

Recently, recognition rate enhancement methods using artificial neural network technology are being studied in signal pattern recognition [20,21,22,23,24]. The existing UWB radar-based methods for recognizing apnea patterns are based on classical machine learning algorithms or on breathing frequency detection [15,16,17,18,19].

Recognition method through detection of respiration frequency can show good performance only when the respiration signal is extracted with a smooth shape and without noise, but if the human motion signal appears similar to the respiration signal, it is difficult to recognize the correct pattern.

If the artificial neural network technology, which has been actively studied recently, is used for breathing pattern recognition, better performance than conventional methods can be expected.

Therefore, in this paper, we propose a novel method to learn and detect five signal patterns of three general respirations (eupnea, bradypnea, and tachypnea), apnea, and body motion using 1-Dimension Convolutional Neural Network (1D CNN). The proposed method constructs an experimental set of neural network parameters and finds the appropriate range of neural network parameters by testing all cases of the set of parameters. And we show the superiority of the proposed method compared with the conventional machine learning algorithms LDA and SVM.

## 2. Related Research

### 2.1. Ultra-Wideband Radar

**Figure 1:**
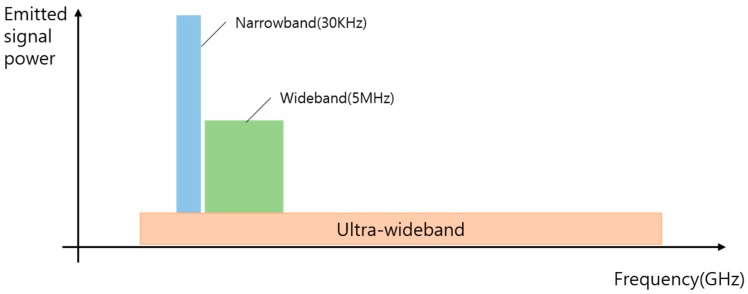
Comparison of the narrowband signal and wideband signal and UWB (Ultra-Wideband) spectrum.

Ultra-WideBand (UWB), which was mainly used for short-range wireless communication, has advantages of low power and wireless transmission speed up to 10 times faster than wireless LAN. As shown in Figure 1, UWB is less likely to interfere with other narrowband or broadband signals. In particular, UWB signals have low spectral power density characteristics over a wide frequency band by using very narrow pulses of several nanometers or pico. Therefore, UWB enables precise measurement of the distance and position of objects, as well as high-speed transmission of data and a high level of security enhancement.

As a result, UWB is attracting attention as a transmission technology to complete the next generation of home networks.

The UWB signal exhibits precision in centimeter units due to the use of a very short pulse and has a very large bandwidth in the low frequency band, so that the transmission characteristic is excellent. Due to these signal characteristics, the UWB radar system can detect obstacles or objects, etc. A UWB radar device is composed of a transmitter and a receiver similar to a general radar system. Each of these devices is comprised of a directional UWB antenna, an impulse signal generator, an impulse detector, a high-speed A/D converter, and a signal processor [25,26,27].

### 2.2. Respiration Signal Patterns

**Figure 2:**
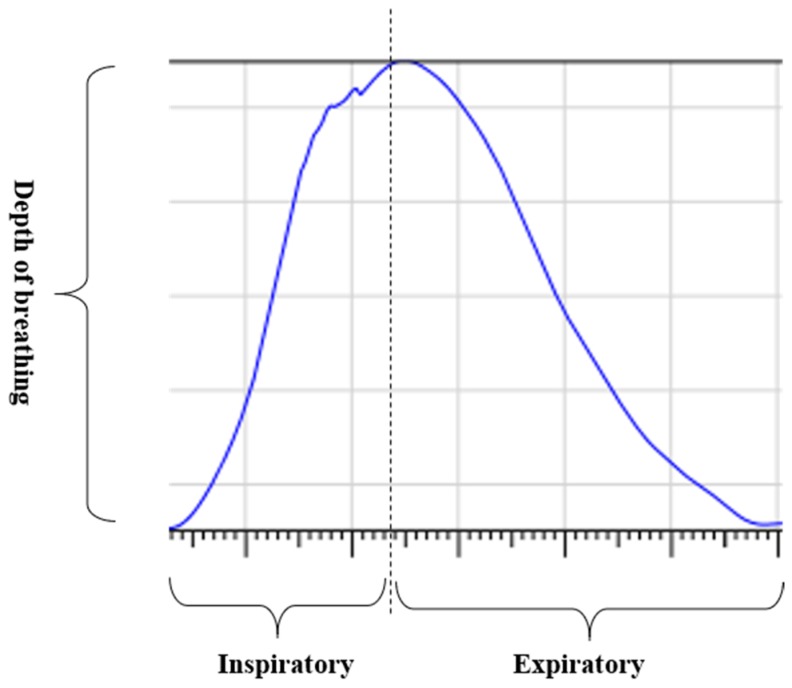
Signal occurred by inspiratory, expiratory, and depth of breathing during breathing.

The respiration signals extracted from UWB Radar appear to rise in the inhalation and fall in the exhalation form as shown in Figure 2. This depends on the state of the person inspiration and expiration, but the ratio of inspiration–expiration is generally 1:1.5 to 1:2 [28,29]. The amplitude of the breathing signal is determined by the degree of deep breathing, and the signal period becomes longer as the length of time of one breath increases.

These breathing signals come in various forms depending on the type of breathing, and are generally defined in four types of breathing, as shown in Table 1, based on the number of breaths per minute of normal breathing. In this paper, the number of breaths per minute for general breathing is defined as 12 to 20 [30,31,32].

### 2.3. Sleep Quality Evaluation Using Respiration

**Figure 3:**
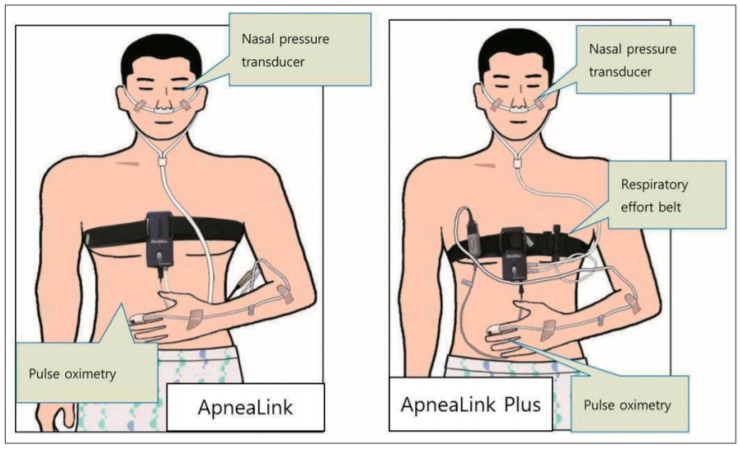
Portable sleep inspection devices “ApneaLink and ApneaLink Plus”.

In general, in the medical field, polysomnography is used to judge whether or not there is sleep apnea, and to check whether there is any disease. A polysomnography measures respiratory disorder, sleep quality, snoring, blood oxygenation, and alertness levels during sleep and provides information on the severity of sleep disordered breathing [1,2]. However, this polysomnography is limited in use because it can only be performed at a facility equipped with a special device that measures limited information. In addition, most of the devices are attached directly to the body, and since the sleep study is done in the facility, it is sometimes difficult to obtain accurate information by measuring other indicators than usual. To solve this problem, a polysomnography through a portable sleep checker as in Figure 3 is also conducted [3,4].

The portable sleep test device should be able to basically measure at least three items of respiratory airflow, respiratory function and blood oxygen saturation level.

The respiratory flow measures the amount of air entering or exiting through the tube and sensor inserted into the nasal cavity during respiration, and the respiratory ability is measured by the belts of changes in the expansion and contraction of the chest according to respiration. These devices are effective for accurate sleep analysis, but they are limited for use in health care applications where devices need to be attached and detached daily. Therefore, to monitor and analyze human sleeping conditions in the healthcare field, it is necessary to utilize minimum equipment, which requires a non-contact sensor.

### 2.4. D Convolutional Neural Network for Signal Pattern Recognition

**Figure 4:**
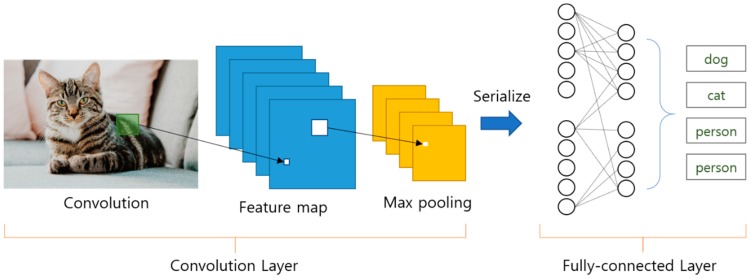
General Convolutional Neural Network structure for image classification.

In general, a CNN (Convolutional Neural Network) is a neural network that has been studied for object detection or recognition based on image data. For this reason, the convolutional layer uses N × M two-dimensional kernel for image feature extraction as shown in Figure 4. Then, the features extracted via convolution of multiple layers are finally connected with the fully-connected layer to execute classification.

Since the respiration signal is not an image but a one-dimensional signal, in order to learn this with the existing CNN, it must be forcibly transformed into two-dimensional data. In such a case, forced data conversion can generate meaningless features, which can lead to a decrease in the performance of the neural network. Therefore, a configuration of Convolutional Neural Network suitable for the characteristics of one-dimensional data is required.

**Figure 5:**
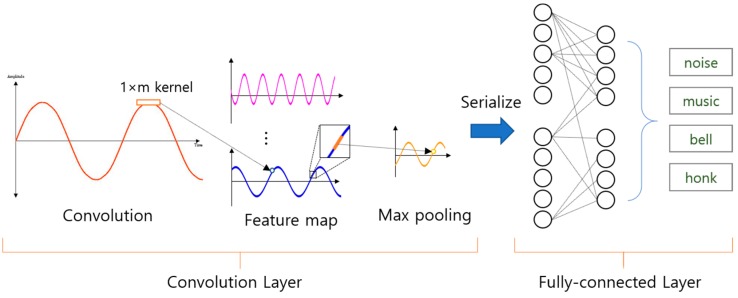
General structure of 1-D Convolutional Neural Network for signal pattern recognition.

Convolutional Neural Network for one-dimensional data learning is shown in Figure 5, the convolution layer receives vector type input and performs convolution using 1 × m kernel. As a result, the feature map is extracted as a one-dimensional data form, so that it can be used for learning respiration signal data. 1D CNN’s convolutional layer also extracts feature maps for classification from input signal data similar to the 2D CNN convolutional layer. The extracted feature maps are composed of fully-connected layer for classification by serializing after Max Pooling process, and its shape is the same as 2D CNN.

1D CNN, which can learn 1D data, has been used in various researches on signal pattern recognition in existing studies. An example is heartbeat signal pattern recognition extracted from an electrocardiogram (ECG) sensor. The 1D CNN method enables various pattern recognition of heart beat signals of ECG sensor. This 1D CNN method shows much higher recognition rate results than SVM or MLP which are traditional machine learning algorithms [33,34]. The structure of the neural network shown in the existing 1D CNN-based signal pattern recognition research uses two or three convolutional layers and fully-connected layers, and shows the classification results in the output layer. In this case, since the recognition rate of the neural network changes depending on the depth and parameters of the neural network, it is very important to maximize the recognition rate of the neural network by setting the optimal parameters. Therefore, in this research, we are attempting to optimize various neural networks through experiments by configuring various parameters for data processing of respiratory signals.

## 3. Proposed Method

### 3.1. Layer Design of 1-D CNN

**Figure 6:**
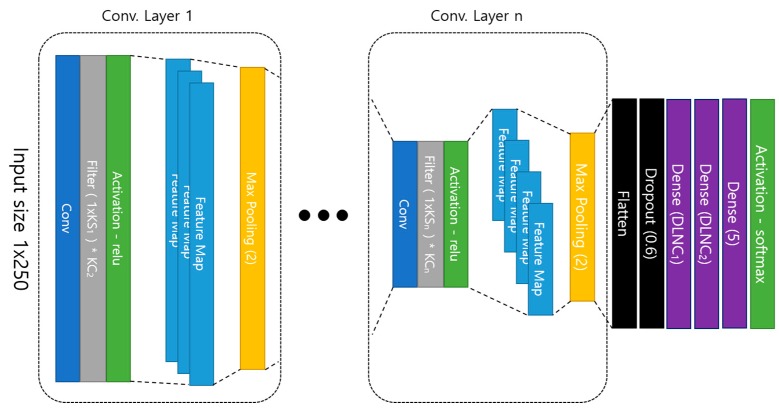
Proposed 1D Convolutional Neural Network structure.

The structure of the proposed 1-D Convolutional Neural Network is shown in Figure 6, and the overall structure is an input layer, a convolutional layer, and a dense layer, and is a general type of a convolutional neural network. Then, the feature map extracted from the last convolution layer is connected to the fully-connected layer by randomly selecting only the specified proportion of features through dropout after being serialized. Dropout is a technique to select only a part of the entire feature at random, which improves performance over all features [33,34].

Therefore, since the features are randomly selected in each learning, the output layer results are derived differently. In this paper, we set the dropout ratio to 0.6 and use only 60% of the features in the learning.

In addition, we use the depth of the convolution layer, the kernel size of each convolution layer, the number of kernels, and the number of neurons of the fully-connected layer as parameters to find the optimal model in the proposed method.

In order to construct the 1D CNN with the maximum recognition rate, experiments are performed by combining various cases of parameters, and as a result, an optimal parameter range is searched.

The algorithm for finding optimal parameters consists of two steps. The first stage algorithm finds the optimal convolutional layer depth and the second stage algorithm finds the optimal parameter range in the depth determined in the first stage. In order to find the optimum parameter range, the experiment is performed N times in the depth determined in step 1, and the parameters in the case of the highest recognition rate per order are included in the optimum parameter range.

Algorithm 1 first constructs a set of experimental parameters to find the depth of the convolutional layer. Then, the accuracy and average accuracy for all cases (*z*) of the experimental parameter set are calculated within the depth range, while increasing the depth of the convolutional layer within the appropriate learning time range. Finally, the Depth value when the average accuracy per depth range is maximum is used as the optimal Depth parameter ($D_{opt}$).

**Algorithm 1** Algorithm for finding optimal convolutional layer depth parameter ($D_{opt}$)
(1)Set the Convolutional Layer Depth parameter ($CLD_{n}$).
$$CLD_{n},\left(CLD_{n}\in CLD,n=1,2,3,\ldots ,T\right)\;when\;CLD=\left\{1,2,3,\ldots ,T\right\}$$(2)Set the Kernel Size ($KS_{j}$).
$$KS_{j},\left(KS_{j}\in KS,j=1,\ldots ,10\right)\;when\;KS=\left\{5,9,13,17,21,25,29,33,37,41\right\}$$(3)Set the Kernel Count ($KC_{k}$).
$$KC_{k},\left(KC_{k}\in KC,k=1,\ldots ,5\right)\;when\;KC=\left\{32,64,128,256,512\right\}$$(4)Set the Dense Layer Neuron Count ($DLNC_{l}$).
$$DLNC_{l},\left(DLNC_{l}\in DLCN,l=1,\ldots ,4\right)\;when\;DLNC=\left\{256,512,1024,2048\right\}$$(5)Define the Convolution Layer parameter combination ($Conv_{n-th}$, n=1,…,T).⊙: combination for all cases in set of elements.$$Conv_{n-th}:\{KS_{j}⊙KC_{k}{\}}_{j=1\ldots 10;k=1\ldots 5}$$(6)Define the Dense Layer parameter ($Dense_{m-th}$, m=1,2).$$Dense_{m-th}:\{DLNC_{l}{\}}_{j=1\ldots 10;k=1\ldots 5}$$(7)Define the $Param_{n-th}$ by the combination (⊙) of the parameters (1), (5) and (6) in Convolutional Layer depth n.$$Param_{n-th}:\{CLD_{n}⊙Conv_{n-th}⊙Dense_{m-th}{\}}_{n=1,2,3;m=1,2}$$(8)The recognition rate for each parameter combination and the average recognition rate at Convolutional Layer Depth n are calculated as follows.Begin Loop:for (*n* = 1; *n* < = T; n++)
①Configures z parameter combinations $CP_{n-th}$ (Combination of Parameters) for all cases on depth n. $$CP_{n-th}:\left\{Param_{n-th}^{1},\ldots ,Param_{n-th}^{z}\right\}$$②Configures the $Acc_{n-th}$ by calculating the recognition rate of each $$Param_{n-th}^{p}\left(p=1,\ldots z\right)\;\mathrm{for}\;\ldots CP_{n-th}.$$
$$Acc_{n-th}:\left\{acc(Param_{n-th}^{1}),\ldots ,acc\left(Param_{n-th}^{z}\right)\right\}$$③Compute average recognition rate $\mu Acc_{n-th}$ and store the result value and depth value *n* to $Result_{n}$.
$$\mu Acc_{n-th}=\frac{1}{p}\left\{\overset{z}{\underset{p=1}{\sum }}acc\left(Param_{i-th}^{p}\right)\right\}$$
$$Result_{n}:\left\{n,\mu Acc_{n-th}\right\}$$
End Loop:(9)Find the maximum $\mu Acc_{n-th}$ of T Results and set the depth value n to the D_opt_.$$D_{opt}=\{n|n\in Result_{n},argmax\left(\mu Acc_{n-th}\right)\}$$


Algorithm 2 performs N iterative learning to find the optimal parameters stabilized in the convolutional layer where the depth is set, and finds the optimum parameter range composed of the parameter values in the case of the highest recognition rate for each learning.

**Algorithm 2** Algorithm for finding optimal range of 1-D CNN parameters
(1)Within optimal depth $D_{opt}$, define ${\mathrm{Param}}_{D_{opt}}$ by combination (⊙) of parameters $Conv_{n-th}$ and $Dense_{m-th}$.
$${\mathrm{Param}}_{D_{opt}}=\left\{D_{opt}⊙Conv_{n-th}⊙Dense_{m-th}\right\}$$(2)On the optimal depth $D_{opt}$, each recognition rate for parameter combinations in all cases is repeated N times as follows.Begin Loop:  for (t = 1; t <= N; t++){①Construct a parameter set ${\mathrm{CP}}_{D_{opt}}$(Combination of Parameters) for the number z in all cases.$${\mathrm{CP}}_{D_{opt}}:\left\{{\mathrm{Param}}_{D_{opt}}^{1},\ldots ,{\mathrm{Param}}_{D_{opt}}^{z}\right\}$$②Configure the ${\mathrm{Acc}}_{D_{opt}}$ by calculating the recognition rate for each ${\mathrm{Param}}_{D_{opt}}^{p}$ (p = 1,…z) in the ${\mathrm{CP}}_{D_{opt}}$.$${\mathrm{Acc}}_{D_{opt}}:\left\{acc({\mathrm{Param}}_{D_{opt}}^{1}),\ldots ,acc\left({\mathrm{Param}}_{D_{opt}}^{z}\right)\right\}$$③Stores in ${\mathrm{Result}}_{t}$ those having the maximum recognition rate among z ${\mathrm{Param}}_{D_{opt}}$.$${\mathrm{Result}}_{t}:\{{\mathrm{Param}}_{D_{opt}}^{p}|{\mathrm{Param}}_{D_{opt}}^{p}\in {\mathrm{CP}}_{D_{opt}},\mathrm{argmax}\left(acc\left({\mathrm{Param}}_{D_{opt}}^{p}\right)\right)\},$$
p = 1,…,z}End Loop:(3)For ${\mathrm{Result}}_{t}$ (t = 1, …, N),Find the optimal parameter ranges (${\mathrm{KS}}_{opt}$, ${\mathrm{KC}}_{opt}$, $DLNC_{opt}$).
$${\mathrm{KS}}_{t}=\{KS|KS\in {\mathrm{Param}}_{D_{opt}}^{p},{\mathrm{Param}}_{D_{opt}}^{p}\in {\mathrm{Result}}_{t}\},$$
$${\mathrm{KC}}_{t}=\{KC|KC\in {\mathrm{Param}}_{D_{opt}}^{p},{\mathrm{Param}}_{D_{opt}}^{p}\in {\mathrm{Result}}_{t}\},$$
$$DLNC_{t}=\{DLNC|DLNC\in {\mathrm{Param}}_{D_{opt}}^{p},{\mathrm{Param}}_{D_{opt}}^{p}\in {\mathrm{Result}}_{t}\}$$
$$\mathrm{Min}({\mathrm{KS}}_{t})<={\mathrm{KS}}_{opt}<=\mathrm{Max}({\mathrm{KS}}_{t})$$
$$\mathrm{Min}({\mathrm{KC}}_{t})<={\mathrm{KC}}_{opt}<=\mathrm{Max}({\mathrm{KC}}_{t})$$
$$\mathrm{Min}({\mathrm{DLNC}}_{t})<={\mathrm{DLNC}}_{opt}<=\mathrm{Max}({\mathrm{DLNC}}_{t})$$


### 3.2. Optimal 1D CNN Parameters

The proposed method differs in performance depending on the combination of CNN parameters. Therefore, the goal is to find a combination of parameters with optimum performance. In order to maximize the recognition rate in the structure of the proposed neural network, we construct a set of parameter values to be optimized as shown in Table 2, and perform the recognition rate test for all cases where the parameter values can be combined. Items of experimental parameters are CLD (convolutional layer depth), KS (kernel size of convolutional layer), KC (kernel count of convolutional layer), and DLNC (neuron count of dense layer). Since there is a limit to test the recognition rate for the combination of all natural numbers that each parameter entry can have, we configure a set of setting values for each item as shown in Table 2 and test the number of all cases in the set.

The data used to find the optimal parameters consist of 1000 data sets of 200 each for each of five respiration patterns, and the ratio of the training set and the validation set is set to 6:4 in the corresponding data set. Recognition rate experiments are conducted for all cases combined by Convolution Layer Depth. The number of values that each parameter can have according to the Convolution Layer Depth (CLD) is as shown in Table 3. The number of cases is calculated by multiplying the number of parameters that each layer can have. The number of cases is 800 when CLD is 1, 40,000 when 2, and 2,000,000 when 3.

**Figure 7:**
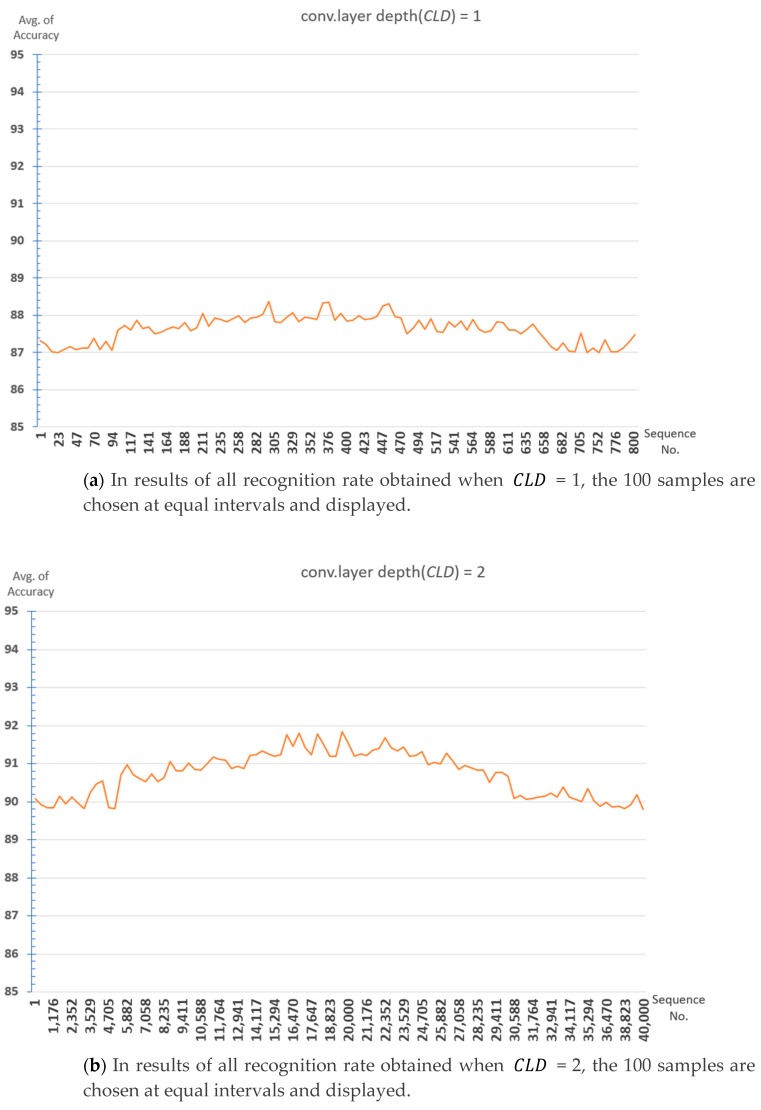

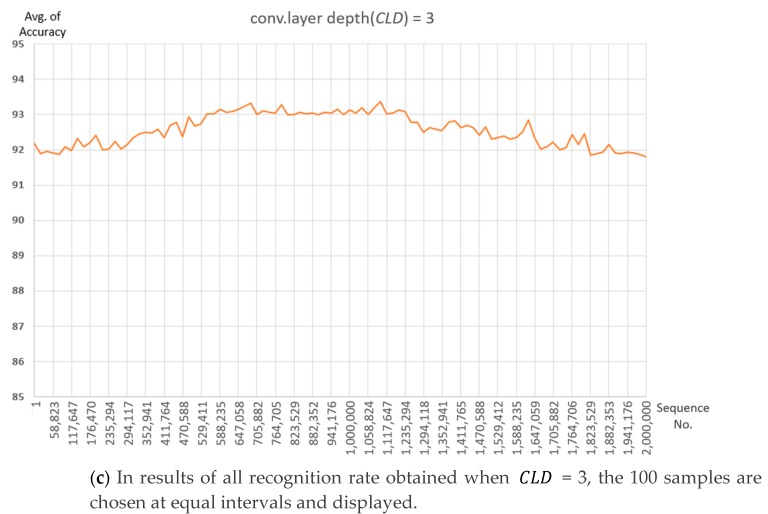
Recognition rate graph according to convolutional layer depth (CLD).

The 100 samples are chosen at equal intervals in results of recognition rate experiments for all cases of parameter combinations for each depth is shown in Figure 7. The average recognition rate at CLD = 1 is about 87.4%, and the average recognition rate at CLD = 2 is about 90.7% and increases by about 3.3%. The recognition rate at CLD = 3 is 92.6%, this case is increased by about 1.9% compared to CLD = 2. It is expected that it will not be possible to expect a large recognition rate improvement even if the number of convolutional layers is further increased. Finding optimal parameters at deeper depths requires too much time for learning due to having too many cases.

**Figure 8:**
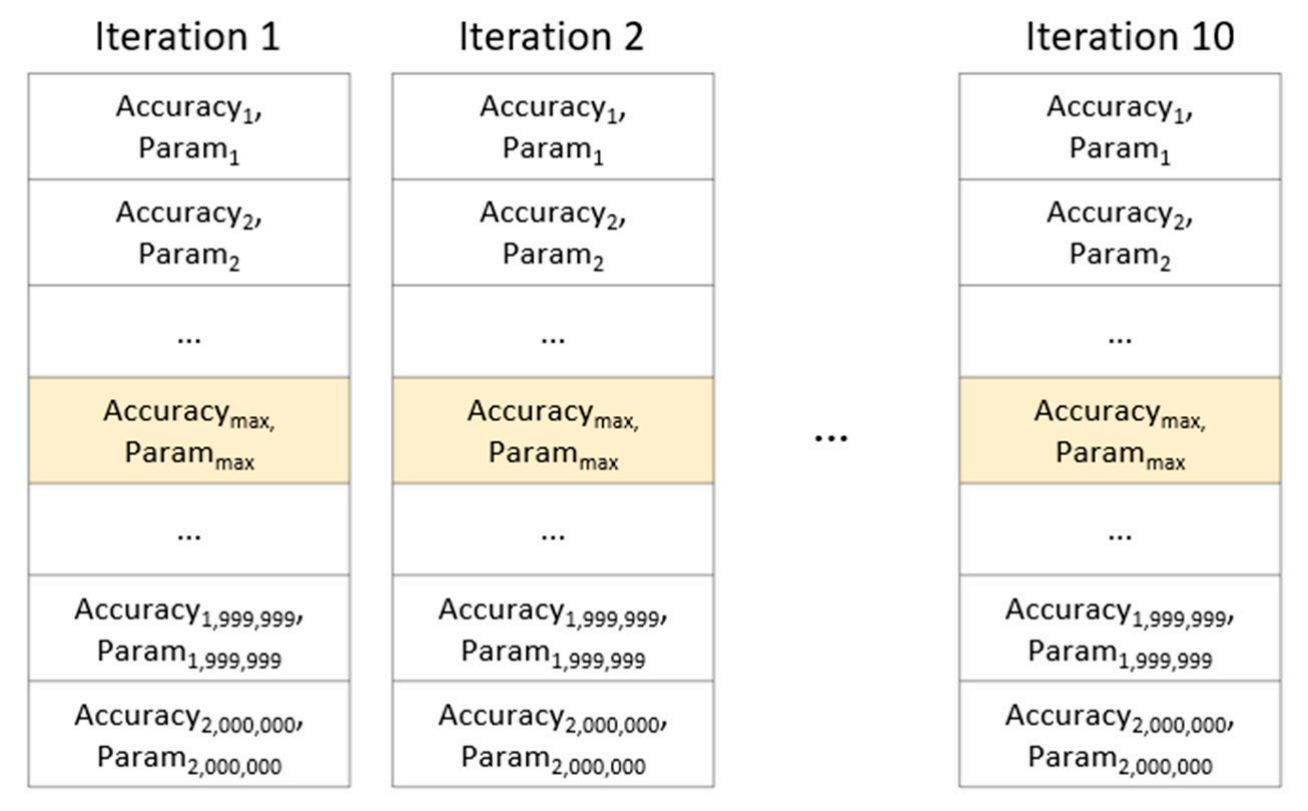
For iterative learning 10 times, find the combination of parameters (Param_max_) with maximum recognition rate (Accuracy_max_) per each iteration.

Therefore, in this paper, we experiment with the combination of parameters for the case where Convolutional Layer depth is 3 ($D_{opt}=3$), and find the optimal parameter. However, in the proposed 1D CNN structure, even if the parameters are the same, the learning result is slightly different each time due to the dropout, so it cannot be fixed with a single parameter. We aim to find the optimal parameter range out of the parameter set configured in Table 2 instead of the single parameter. In this paper, in order to obtain this range, the optimal depth of convolutional layer is set to 3 ($D_{opt}=3$), and a total of 10 iterations are performed (N = 10). At this time, to find the optimal parameter range for each iteration as in Figure 8, a parameter set ${\mathrm{Param}}_{D_{opt}}^{p}\left(p=1,\ldots ,z\right)$ to extract the maximum value out of results of 2 million recognition rates is used.

**Figure 9:**
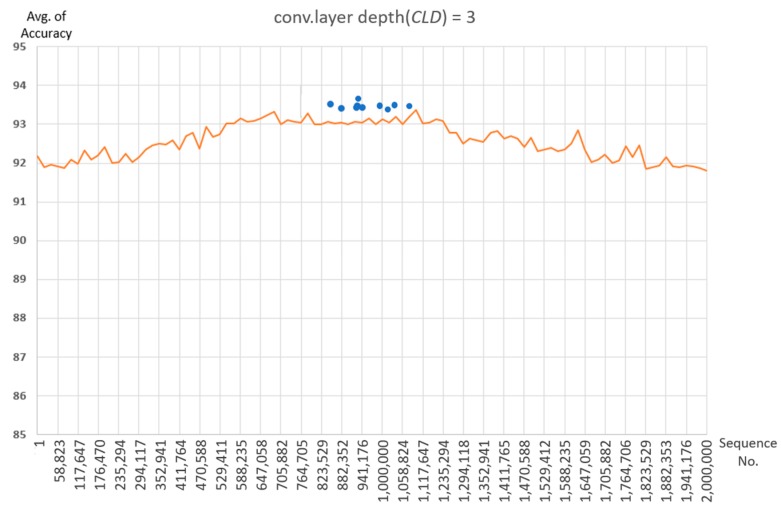
Out of the results of 10 times repeated learning on the CLD = 3, the points where the highest recognition rate per repetition time are located are marked.

In addition, the point to represent the highest recognition rate for each of the 10 repeated learning results are shown in Figure 9.

Figure 9 shows that the training results with a combination of experimental parameters in Table 2 forms a gentle Gaussian graph and shows that the points with the highest recognition rate in the center are concentrated. The 10 parameter sets showing the highest recognition rate results in Figure 9 are the same as Table 4. At this time, the item showing the highest accuracy shows 93.76% accuracy from the 8th Iteration.

We can derive the optimal ranges as shown in Table 5 by taking optimal kernel size ($KS_{opt}$) and kernel count ($KC_{opt}$) of convolutional layer and optimal neuron count of dense layer ($DLNC_{opt}$) from Table 4. If we apply the parameters selected in Table 5 (range of optimal parameters) in the proposed neural network, we can expect an average accuracy of 93.57%.

## 4. Experiments

### 4.1. Data Gathering Environment

The respiration data were collected using UWR (Ultra-Wideband Radar) with the specifications shown in Table 6 below (All subjects gave their informed consent for inclusion before they participated in the study. The study was conducted in accordance with the Declaration of Helsinki, and the protocol was approved by the Ethics Committee of GRRC-Gachon2017(B02)). The device was connected to the PC via UART Serial and data was collected using a program for the measurement of respiratory rate per minute and the storage of respiratory signal data.

**Figure 10:**
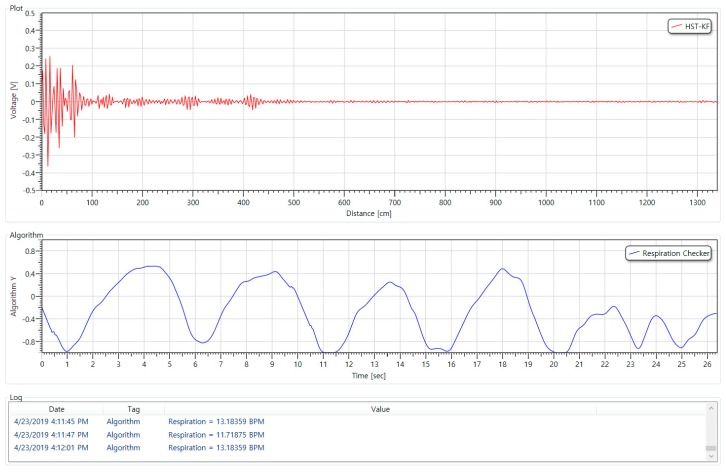
UWB Radar PC program for extracting respiratory signal data.

Results of an executing program to measure the respiratory rate per minute and store the respiratory signal data are shown in Figure 10, there is an area of the screen for indicating a raw signal or a filtered signal at the upper part, and an area for indicating a respiration signal at the center. The area of the screen for indicating respiration rate per minute, calculated by the respiration signal, is located at the lower part.

In the display area of the raw and filtering signals, the amount of the signal reflected back at each distance or the signal to which the raw signal filtering is applied is displayed. In this paper, Kalman filter technique was applied to remove the noise of the raw signal, and the parameters applied to Kalman filter used 0.01 and 0.1 as default settings. Also, in order to extract the respiration signal, a distance value between the sensor and human is required. In this experimental environment, the distance between the sensor and the human thorax was set to 20 cm.

**Figure 11:**
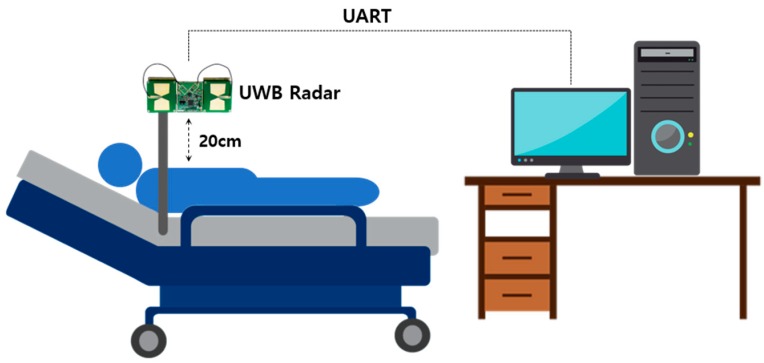
UWB Radar installation environment for collecting respiratory signal data.

The equipment for collecting the respiration signal is shown in Figure 11.

We place a UWB Radar device at a distance of 20 cm from the chest of a person while the person is lying on the bed in a normal state and collect signals for five breathing patterns from the device. The respiratory rate per minute is displayed in the bottom area of the program when collecting respiratory signal data. At this time, the signals of eupnea, bradypnea, tachypnea, and apnea are classified based on respiration rate per minute. In this experimental environment, four patterns of breathing signal and motion signal data were intentionally generated and collected within the range of breathing per minute while awake because of the limitation that it is difficult to collect all patterns of data when a person is sleeping.

### 4.2. Learning and Test Dataset

**Figure 12:**
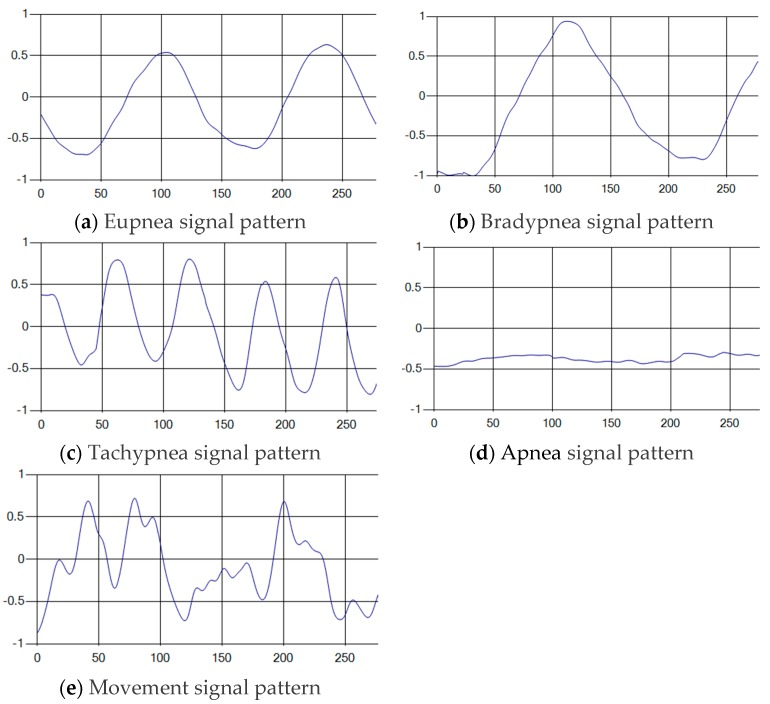
Five signal patterns for learning.

The patterns of UWB respiration signals collected for learning are shown in Figure 12. Eupnea, Bradypnea, and Tachypnea are similar in shape to each other, but it can be seen that signal intensity and cycle are different. Apnea and movement patterns can be noticeable when compared to other breathing patterns.

**Figure 13:**
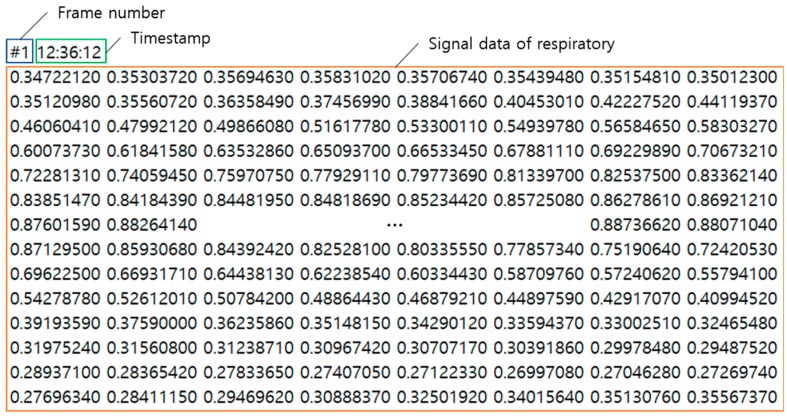
Breathing signal data extracted through UWB Radar.

The structure of the respiration signal data is stored by the program as frame number, time stamp, and respiration signal data, as shown in Figure 13, and the number of data items stored at one point is 660. The UWB Radar used in the experiment generates data at 25 frames per second. In order to acquire experimental data to be used for learning, about 15,000 data items are stored by measuring 10 min for each pattern.

**Figure 14:**
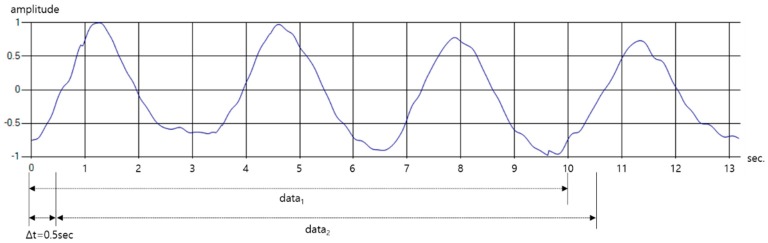
Construction of learning data by various time-shifting in one breathing pattern.

The stored data is not used immediately for learning but after the data length is processed. In general, the device to detect respiratory state in polysomnography measures respiratory amplitude for at least 10 s to distinguish a state among respiratory signals [35]. Therefore, only 250 pieces of data, which is 10 s long, are used from the total of 660 pieces of data. Also, as shown in Figure 14, time-shifting is performed every 0.5 s on one breathing pattern data to form learning data sets of various shapes. Through this process, the learning and testing datasets are organized so that only one pattern is included in one data. Finally, 500 data sets are configured for each breathing pattern.

In addition, the number of people who participated in the data collection totaled 10, and each target’s age, height, weight, and five patterns of respiratory signal data were collected as shown in Table 7. And the total experimental data were collected by a total of 250 data per participant, 50 for each pattern. Thus, the total data is composed of 2500. The age group of volunteers is in their mid-20 s to early 40 s, who are roughly in the range of the average to obese South Korean physique.

### 4.3. Comparison of Accuracy with Other Recognition Methods

**Figure 15:**
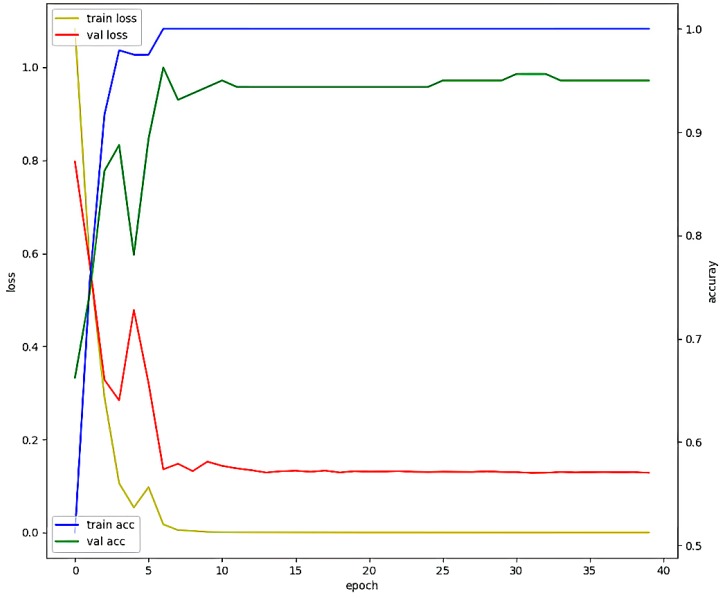
The results of performance obtained by using the optimal parameters of the proposed 1 D CNN model.

To test the proposed 1D CNN model, we set the values of parameters as shown in Table 8 by selecting the parameters in the parameter range of Table 5. The result of learning each breathing pattern is shown in Figure 15. At the time of learning, Epoch is set to 40, batch size is set to 10, and learning is almost completed at Epoch 15. It can be seen that there is almost no fluctuation of accuracy and loss. After learning was completed, the final validation accuracy was 0.948, and the validation loss was 0.183.

**Figure 16:**
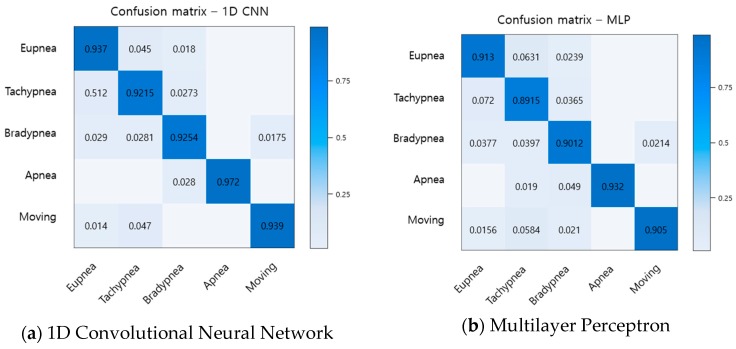

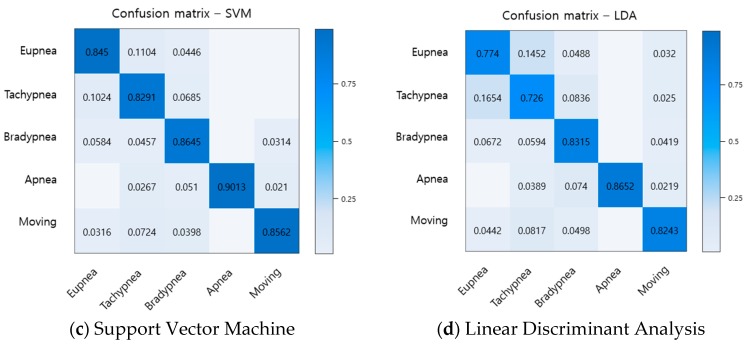
Comparison of recognition rate for breathing pattern among the proposed method and the traditional methods.

To evaluate the performance of the proposed method, the recognition rates of respiratory patterns were compared with traditional machine learning algorithms LDA, SVM, and MLP. The data set used for performance evaluation uses a total of 2500 data items, 500 for each breathing pattern. Of the total 2500 data items, 1500 will be used for learning, and the remaining 1000 will be used for performance testing. As a result, the recognition rates of the proposed method and the conventional method are shown in a confusion matrix as shown in Figure 16.

When comparing the recognition results for each breathing pattern, it was found that the conventional method similarly recognizes eupnea, bradypnea, and tachypnea, and the recognition rate is lower than that of the proposed method. Comparing with the average recognition rate, the LDA was about 80.4%, the SVM was about 86%, the MLP was about 90.9%, and the proposed method was about 93.9%, showing an improvement in the recognition rate from at least 3% to up to 13.5%.

## 5. Conclusions

In this paper, to analyze the quality of sleep, we extracted respiration signals of human using UWB Radar device with non-contact sensor and classify respiratory of five types by proposed method (respiratory pattern recognition algorithm based on 1D Convolutional Neural Network) from extracted respiration signals.

Previous studies using respiration data from UWB radar devices include only apnea signal recognition or measurements of respiratory rate per minute. However, for accurate sleep analysis, recognition of not only apnea but also various other breathing patterns should be collected. In the proposed method, we designed the 1D CNN based learning model to recognize and classify the signal patterns of 5 types for eupnea, bradypnea, tachypnea, apnea, and motion signal, and found the range of optimum parameters to use in the model by executing various experiments. The proposed method could improve the breathing pattern recognition rate from minimum 3% to maximum 13.5% than conventional method.

Therefore, the proposed method can detect not only simple apnea but also other various sleep patterns, so it is expected that it can be used for analyzing respiratory disorders such as bradypnea and tachypnea.

## Figures and Tables

**Table 1:** Types of respiration defined by using breath rate per a minute in medical field.

Respiration Type	Definition and Property of Respiration
Eupnea	→A type of respiration when the breathing rate per minute is 12 to 20 on average by adult male standard.
Bradypnea	→A type of respiration with a breathing rate of 12 breaths per minute or less. When compared with general breathing, the depth of inhalation and exhalation is lowered, and the breathing cycle is increased. It is often observed when sleeping and may be caused by disease.
Tachypnea	→A type of shallow breathing with a breathing rate of 20 breaths per minute or more. It can occur in the presence of illness such as fever, weakness, or mental instability. At normal times, it may occur during light exercise.
Apnea	→Breathing which shows a reduction of more than 90% of the normal respiratory flow for at least 10 s during sleep. This causes the amplitude to be very small in the respiration signal.

**Table 2:** The parameters used in the proposed method.

Parameter Symbol	Parameter Description	Set of Parameters
CLD	- Convolution Layer Depth	{1, 2, 3}
KS	- Kernel size of conv. layer	{5, 9, 13, 17, 21, 25, 29, 33, 37, 41}
KC	- Kernel count of conv. layer	{32, 64, 128, 256, 512}
DLNC	- Neuron count of dense layer	{256, 512, 1024, 2048}

**Table 3:** Number of parameter combination cases according to Depth of Convolutional Layer (CLD ).

Depth of Conv. Layer (CLD)	1st Conv. Layer		2nd Conv. Layer		3rd Conv. Layer		1st Dense Layer	2nd Dense Layer	Number of cases (z)
	Kernel Size	Kernel Count	Kernel Size	Kernel Count	Kernel Size	Kernel Count	Neuron Count	Neuron Count	
1	10	5	-	-	-	-	4	4	800
2	10	5	10	5	-	-	4	4	40,000
3	10	5	10	5	10	5	4	4	2,000,000

**Table 4:** For 10 times repeated learning, the combination of parameters having the maximum recognition rate for each iteration.

Iteration No.	Sequence No.	Parameter	Avg. Of Accuracy
1	942,110	Conv. Layer 1-KS:21, KC:256 Conv. Layer 2-KS:25, KC:128 Conv. Layer 3-KS:29, KC:64 Dense Layer 1-DLNC:2048 Dense Layer 2-DLNC:1024	$acc({\mathrm{Param}}_{D_{opt}}^{942110})$ = 93.51%
2	898,926	Conv. Layer 1-KS:21, KC:128 Conv. Layer 2-KS:21, KC:256 Conv. Layer 3-KS:29, KC:128 Dense Layer 1-DLNC:2048 Dense Layer 2-DLNC:1024	$acc({\mathrm{Param}}_{D_{opt}}^{898926})$ = 93.48%
…			
8	937,339	Conv. Layer 1-KS:21, KC:256 Conv. Layer 2-KS:21, KC:64 Conv. Layer 3-KS:29, KC:256 Dense Layer 1-DLNC:1024 Dense Layer 2-DLNC:2048	$acc({\mathrm{Param}}_{D_{opt}}^{937339})$ =93.76%(maximum)
…			
10	1,098,106	Conv. Layer 1-KS:25, KC:128 Conv. Layer 2-KS:21, KC:128 Conv. Layer 3-KS:29, KC:64 Dense Layer 1-DLNC:1024 Dense Layer 2-DLNC:1024	$acc({\mathrm{Param}}_{D_{opt}}^{1098106})$ =93.58%
Average			93.57%

**Table 5:** Experimental parameters and optimization parameter range of proposed 1-Dimension Convolutional Neural Network (1D CNN) model.

Parameter	Rage of Test Parameters	Range of Optimal Parameters
Convolution layer depth (CLD)	{1, 2, 3}	$CLD\left(D_{opt}\right)$ = {3}
Kernel size (KS)	{5, 9, 13, 17, 21, 25, 29, 33, 37, 41}	$KS_{opt}$ = {21, 25, 29}
Kernel Count (KC)	{32, 64, 128, 256, 512}	$KC_{opt}$ = {64, 128, 256}
Dense Layer Neuron Count (DLNC)	{256, 512, 1024, 2048}	$DLNC_{opt}$ = {1024, 2048}

**Table 6:** UWB Radar Specification used for breathing signal data collection.

Item	Specific
Detecting Range	10~22 m
Frequency Range	3.0~4.0 GHz
Bandwidth	0.45~1.0 GHz
Distance Resolution	1.5~3.3 cm
Antenna Angle	50° (X-Z plane)~77.5° (Y-Z plane)

**Table 7:** Data provider’s body information and five respiration patterns.

Volunteer	Age	Height	Weight	Respiration Signals	
No.1	24	171 cm	70 kg	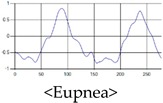	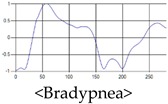
				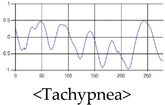	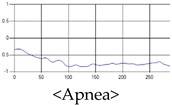
				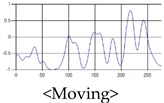	
No.2	25	172 cm	68 kg	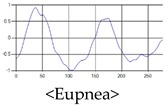	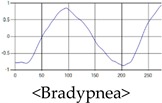
				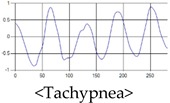	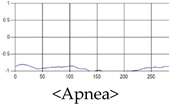
				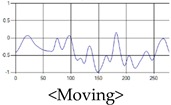	
No.3	27	181 cm	80 kg	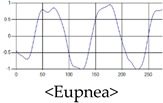	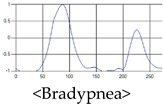
				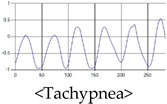	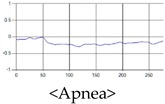
				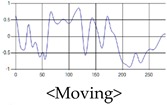	
No.4	27	191 cm	110 kg	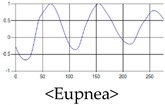	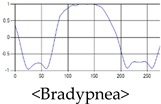
				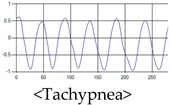	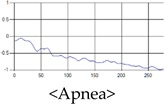
				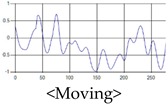	
No.5	30	180 cm	95 kg	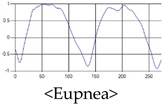	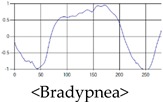
				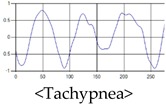	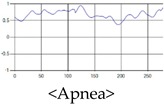
				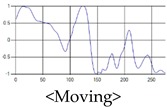	
No.6	31	175 cm	71 kg	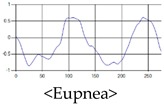	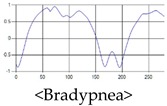
				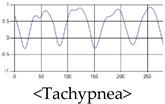	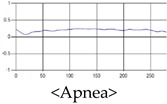
				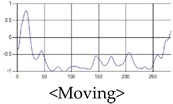	
No.7	34	173 cm	73 kg	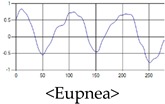	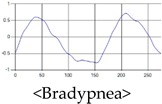
				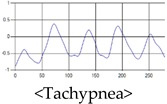	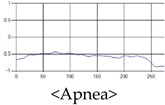
				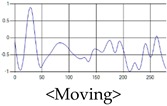	
No.8	34	172 cm	75 kg	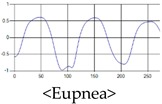	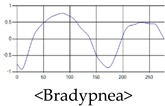
				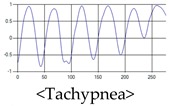	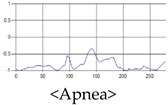
				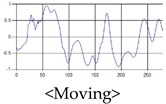	
No.9	35	175cm	113kg	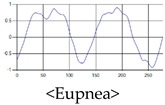	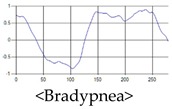
				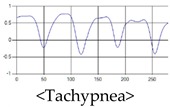	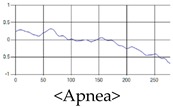
				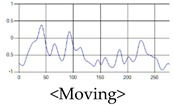	
No.10	41	176cm	79kg	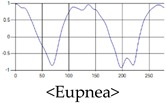	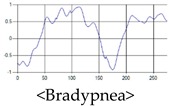
				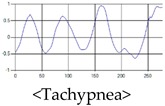	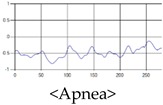
				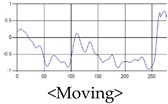	

**Table 8:** To evaluate the performance of the proposed 1-D CNN model, each parameter value is selected from the optimal parameter range.

Parameter	Used Values
Convolution layer depth (CLD)	3
Kernel size (KS)	Conv. Layer 1:29
	Conv. Layer 2:25
	Conv. Layer 3:21
Kernel count (KC)	Conv. Layer 1:64
	Conv. Layer 2:64
	Conv. Layer 3:128
Dense layer neuron count (DLNC)	Dense Layer 1:2048
	Dense Layer 2:1024
